# Lipid Metabolism Influence on Neurodegenerative Disease Progression: Is the Vehicle as Important as the Cargo?

**DOI:** 10.3389/fnmol.2021.788695

**Published:** 2021-12-20

**Authors:** Raja Elizabeth Estes, Bernice Lin, Arnav Khera, Marie Ynez Davis

**Affiliations:** ^1^VA Puget Sound Health Care System, Seattle, WA, United States; ^2^Division of Biological Sciences, University of Montana, Missoula, MT, United States; ^3^Department of Neurology, University of Washington, Seattle, WA, United States

**Keywords:** lipid metabolism, protein aggregation and propagation, extracellular vesicle, Parkinson’s disease, Alzheimer’s disease, glia, glucocerebrosidase (GBA), ceramide

## Abstract

Many neurodegenerative diseases are characterized by abnormal protein aggregates, including the two most common neurodegenerative diseases Alzheimer’s disease (AD) and Parkinson’s disease (PD). In the global search to prevent and treat diseases, most research has been focused on the early stages of the diseases, including how these pathogenic protein aggregates are initially formed. We argue, however, that an equally important aspect of disease etiology is the characteristic *spread* of protein aggregates throughout the nervous system, a key process in disease progression. Growing evidence suggests that both alterations in lipid metabolism and dysregulation of extracellular vesicles (EVs) accelerate the spread of protein aggregation and progression of neurodegeneration, both in neurons and potentially in surrounding glia. We will review how these two pathways are intertwined and accelerate the progression of AD and PD. Understanding how lipid metabolism, EV biogenesis, and EV uptake regulate the spread of pathogenic protein aggregation could reveal novel therapeutic targets to slow or halt neurodegenerative disease progression.

## Introduction

Age-related neurodegenerative diseases are a growing medical burden in our aging population. Alzheimer’s disease (AD), the most common neurodegenerative disease, affects 11.3% of people ages 65 or older, and Parkinson’s disease (PD), the second most common neurodegenerative disease, affects approximately 1% of people ages 60 or older. Therapies for these diseases are limited to symptomatic treatments only, and there is an urgent need for treatments that could halt or slow the underlying neurodegenerative processes.Many neurodegenerative diseases, including AD and PD, have characteristic neuropathologic findings of pathologic bodies that include proteins specific to each disease. In AD, amyloid β-peptides (Aβ) formed by proteolytic cleavage of the amyloid precursor protein (APP) by secretases aggregate into extracellular amyloid plaques, and hyperphosphorylated tau accumulates intracellularly to form neurofibrillary tangles (NFTs; Masters et al., 1985; Grundke-Iqbal et al., 1986). PD and dementia with Lewy Bodies (DLB) are characterized by Lewy bodies (LBs) and Lewy neurites (LN) that include oligomerized α-synuclein (α-syn), as well as lipid membranes and organelles (Spillantini et al., 1997; Shahmoradian et al., 2019). While we will focus on AD and PD-related pathology in this review, several other neurodegenerative diseases such as other α-synucleinopathies and tauopathies, Huntington’s disease, Amyotrophic lateral sclerosis (ALS), and Prion disease are also characterized by neuronal inclusions with specific pathologic protein aggregates. Understanding the mechanisms underlying protein aggregation and spread in AD and PD could hopefully be applied to other aggregate-prone neurodegenerative conditions to develop disease-modifying therapies.

Each neurodegenerative pathologic aggregate has a distinctive temporo-spatial pattern of spread throughout the nervous system that is characteristic of the neurodegenerative disease and correlates with clinical progression (Bancher et al., 1993). AD disease onset is associated with the formation of Aβ plaques and NFTs in the entorhinal cortex, with predictable spread to the hippocampus and eventually throughout the neocortex as the disease progresses (Braak and Braak, 1991; Braak et al., 2006; Nath et al., 2012). In PD, LBs and LNs first appear in the lower brainstem and olfactory neurons, ascending through the midbrain and limbic system to eventually spread throughout the neocortex by the end-stage of the disease (Braak et al., 2003). Understanding the mechanisms regulating the spread of these neurodegeneration-associated aggregates, which are closely associated with clinical disease progression, could reveal novel pathways that could be therapeutically altered to slow or halt disease progression.

A major clue into the mechanism of protein aggregate spread emerged from early investigations of GBA-associated PD. *Glucosylceramidase Beta* (*GBA*) encodes a lysosomal enzyme glucocerebrosidase (GCase), that hydrolyzes glucosylceramide into glucose and ceramide. Recessive mutations in *GBA* cause Gaucher’s disease, the most common lysosomal storage disease [*GBA* (OMIM 606463)]. However, in the early 1990s, clinicians caring for Gaucher’s patients noted that there seemed to be an unusually high prevalence of PD in 1st degree relatives, far greater than expected within the general population (Neudorfer et al., 1996). Through further clinical and genome- wide association studies, *GBA* mutations are now recognized to increase the risk of developing PD by at least 5-fold compared to non-carriers making *GBA* mutations the strongest genetic risk factor for PD (Sidransky et al., 2009; Nalls et al., 2013). Examination of longitudinal cohorts of PD patients later revealed that *GBA* mutations not only increase the risk of developing PD but also lead to a more malignant clinical course. PD patients carrying *GBA* mutations have an increased risk of developing cognitive deficits and faster progression of both cognitive and motor function decline (Winder-Rhodes et al., 2013; Brockmann et al., 2015; Davis et al., 2016a, b). This observation was further supported by a transgenic α-syn^A53T^ mouse model that had an earlier onset and faster progression of degenerative symptoms in a heterozygous GBA knockout background (Tayebi et al., 2017). These findings suggest that *GBA* may influence not only the development of Lewy pathology in PD but also the spread of Lewy pathology throughout the brain, manifested by accelerated disease progression.

Increasing evidence suggests that extracellular vesicles (EVs) such as exosomes and synaptic vesicles (SVs) play a major role in the propagation of neurodegeneration-associated protein aggregates throughout the brain. Crucially, examination of *GBA*-PD patient tissues as well as modeling *GBA*-associated neurodegeneration has revealed that GBA activity regulates EVs, which may explain its role in accelerating protein aggregate spread (Papadopoulos et al., 2018; Thomas et al., 2018; Jewett et al., 2021). As *GBA* encodes an enzyme important for ceramide metabolism, investigations into the lipid metabolism alterations due to *GBA* mutations have led to interesting hypotheses into new mechanisms by which lipid metabolism influences neurodegenerative disease progression. Lipid and in particular ceramide metabolism alterations have also been reported in AD (Han et al., 2002; Wang et al., 2008; Mielke et al., 2010, 2012; Czubowicz et al., 2019), supporting a role for ceramide in promoting aggregation of Aβ associated with lipid rafts and spread of tau and amyloid beta (Aβ) *via* exosomes, as well as alterations in lipid metabolism associated with disease risk and progression. In this review, we will next examine the evidence supporting the role of lipid metabolites in influencing exosome biogenesis, uptake by recipient cells, and rate of disease progression. Together, these suggest that the vehicle, not just the cargo of EVs, is important for the pathogenesis of neurodegenerative diseases.

## Lipid Metabolism in Neurodegeneration

Lipids are crucial for the normal development and function of the central nervous system (CNS). Sphingolipids and cholesterol are the most abundant class of lipids that are found in the CNS, mainly residing in myelin (O’brien and Sampson, 1965; Kishimoto et al., 1969; Giussani et al., 2021). Lipids are not only the building blocks for membranes, but lipid composition, degree of unsaturation, and length of fatty acyl tails can dictate fluidity of membranes, lipid raft membrane microdomains, and influence vesicle fusion and secretion (Mencarelli and Martinez-Martinez, 2013). Sphingosine, a metabolite of ceramide, is also bioactive and can regulate multiple cellular functions, including signaling, apoptosis, mitochondrial function, immune function, and metabolism (Mencarelli and Martinez-Martinez, 2013; Wang and Bieberich, 2018).

Lipids are increasingly implicated in neurodegenerative diseases. Identification of a growing number of genes involved in lipid metabolism through Mendelian inheritance, genome-wide association studies (GWAS), and transcriptomic studies have been implicated in AD, PD, and other neurodegenerative diseases. The *Apolipoprotein epsilon4* allele is the most common genetic risk factor for AD and is important for transporting cholesterol into the brain (Mahley, 1988; Huang and Mahley, 2014). Numerous lipid metabolism-related risk genes have been identified through GWAS studies for AD, recently reviewed by Chew et al. (2020). A growing number of causative genes and risk loci for PD have also been implicated in lipid metabolism, including *PLA2G6/PARK14, SCARB2, SMPD1, SREBF1, DGKQ*, which were recently reviewed (Alecu and Bennett, 2019). And, as reviewed above, *GBA* is a critical gene in ceramide metabolism, and mutations in *GBA* are the most penetrant genetic risk factor for PD.

Lipidomic analysis of tissues from AD patients and AD animal models have revealed alterations in the levels of numerous lipids, including fatty acids, glycerolipids, glycerophospholipids, sphingolipids, and cholesterol (Mesa-Herrera et al., 2019; Chew et al., 2020). Phospholipid deficiencies such as the level of ethanolamine plasmalogen relative to phosphatidylethanolamine levels were observed in postmortem brain tissue of AD patients (Ginsberg et al., 1995). Ceramide has been of particular interest given the paradigm of the ceramide-sphingosine-1-P (S-1-P) rheostat where the balance of these two closely linked and regulated lipids may determine cell death vs. survival (reviewed in Hait et al., 2006; Taniguchi and Okazaki, 2020). While ceramide has been shown to have pro-apoptotic, autophagic, and inflammatory effects, S-1-P has a pro-survival effect mediated by G-protein coupled receptor signaling. Ceramide is also implicated in AD pathogenesis by promoting aggregation of Aβ through interaction *via* lipid rafts, which are enriched in cholesterol and sphingolipids, and ceramide-enriched exosome membranes (Czubowicz et al., 2019). Higher ceramide levels have been reported in several studies analyzing tissue from AD patients and rodent models of AD. Brain tissue from AD patients with mild to moderate symptoms associated with higher total ceramide levels compared to age-matched controls (Han et al., 2002). Ceramide levels were also increased in cerebrospinal fluid (CSF) of AD patients compared to ALS patients and controls, and immunohistochemistry of frontal cortex revealed increased ceramide in astrocytes (Satoi et al., 2005). Analysis of plasma from a small group of individuals with AD, mild cognitive impairment, and controls found that higher baseline levels of the long-chain ceramides C22:0 and C24:0 were predictive of cognitive decline and hippocampal atrophy (Mielke et al., 2010). Analysis of serum in a longitudinal study of 99 women during their 8th decade again found higher baseline levels of long-chain ceramides to be associated with increased risk of AD (Mielke et al., 2012). Serum ceramide levels were increased in both AD and DLB patients (Savica et al., 2016). Ceramides have also been found to be increased in brain tissue from rodent models of AD (Alessenko et al., 2004; Wang et al., 2008).

Significant alterations in lipid metabolism are also observed in PD. While it is not unexpected that *GBA* carriers with PD may have lipid metabolism abnormalities, even sporadic PD patients without *GBA* mutations were observed to have reduced GCase activity in postmortem tissue (Murphy et al., 2014). Furthermore, the level of reduction in *GBA* enzyme activity inversely correlated with α-syn expression, although high molecular weight α-syn oligomers were not assessed in this study (Murphy et al., 2014). Subsequent studies have confirmed that even sporadic PD patients without *GBA* mutations have reduced GCase enzyme activity, albeit to a lesser extent than GBA carriers with PD (Alcalay et al., 2015). Sporadic PD patients also have altered levels of glucosylceramide and ceramide, the substrate and product of GCase enzyme activity (Mielke et al., 2013; Guedes et al., 2017), further supporting the observation that GBA enzymatic dysfunction is not a prerequisite for ceramide accumulation. EVs isolated from PD patient brain tissue were found to have increased levels of membranous ceramides that increased binding affinity for α-syn, leading to increased α-syn accumulation, aggregation, and propagation (Kurzawa-Akanbi et al., 2021). These studies suggest that reduced GCase activity and the resulting alterations in lipid metabolism are critical in the pathogenic process causing PD. Finally, reducing the substrate of GCase in a mouse model of *GBA* PD through inhibition of glucosylceramide synthase reduced insoluble α-syn oligomerization and accumulation of ubiquitinated proteins (Sardi et al., 2017), providing compelling evidence that ameliorating altered lipid metabolism in PD can reduce the underlying pathology.

Ceramides have multiple bioactive influences, and it remains unclear whether they may have a neuroprotective or neurotoxic influence. Since *GBA* mutations lead to accumulation of glucosylceramide and possible relative reduction in ceramide, it has been hypothesized that reduced ceramide may also contribute to the pathogenesis in *GBA*-related PD. In support of this hypothesis, GCase-deficient HEK293 cells treated with exogenous C18-Ceramide or the acid ceramidase Carmofur reversed impairment in secretory autophagy and reduced α-syn levels (Kim et al., 2018). Similar findings were observed in iPSC-derived dopaminergic neurons heterozygous for a *GBA* mutation, where treatment with Carmofur resulted in reduced ubiquitinated proteins and oxidized α-syn species (Kim et al., 2018). Treatment of our *Drosophila* *GBA* deficient model with Carmofur also led to a reduction in accumulated insoluble ubiquitinated proteins (unpublished).

Altered metabolism of ceramide and glucosylceramide leads to additional downstream lipid alterations with as yet unclear pathogenic effects. In a double mutant transgenic mouse model heterozygous for *GBA* and expressing wild-type human α-syn through its endogenous promoter, glucosylsphingosine was significantly increased, while glucosylceramide, ceramide, and sphingosine levels were similar to wild-type *GBA* controls without human α-syn expression (Ikuno et al., 2019). This is likely due to the deacylation of glucosylceramide by lysosomal acid ceramidase, resulting in glucosylsphingosine, which also accumulates in Gaucher’s disease (Ferraz et al., 2016). Glucosylsphingosine can exit the lysosome, where it can be hydrolyzed by non-lysosomal GBA2 into ceramide, sphingosine, and sphingosine-1-phosphate (Abed Rabbo et al., 2021), resulting in complex alterations of downstream lipids that remain poorly understood.

Phospholipid bis(monoacylglycerol)phosphate (BMP) species are also lipids of interest in neurodegeneration, as they are enriched in late endosome-lysosomes. Inhibiting Vps34, a lipid kinase important for the synthesis of phosphatidylinositol-3-phosphate (PI3P), which was found to be reduced in the AD brain and is an important regulator of endosomal trafficking (Morel et al., 2013), resulting in increased secretion of exosomes by cultured neurons and in a conditional knockout mouse model (Miranda et al., 2018). The increased exosome biogenesis occurred in the context of lysosomal stress, where C-terminal APP fragments were selectively sorted as exosome cargo. The resulting exosomes had a unique enrichment in BMPs in addition to ceramides and sphingomyelin (Miranda et al., 2018). Interestingly, specific urine BMPs were found to be associated with *LRRK2* G2019S mutation carriers with PD and cognitive decline (Alcalay et al., 2020). *LRRK2*, the most common genetic cause of PD, is also implicated in endolysosomal function (Erb and Moore, 2020), suggesting BMPs could be exploited as a biomarker for lipid alterations associated with neurodegeneration involving endolysosomal impairment.

Another way that lipid metabolism alterations may promote neurodegenerative pathology is through direct interaction with aggregate-prone proteins. Significant evidence supports this to be true with α-syn. Sharon et al. (2003) demonstrated that α-syn oligomerizes into insoluble aggregates in the presence of polyunsaturated fatty acids, but not saturated fatty acids, and the length of the fatty acyl chain also influences oligomerization. In an *in vitro* system using small unilamellar vesicles, α-syn adopted an amyloid-like conformation under conditions where it binds to the lipid bilayer (Galvagnion et al., 2015). In AD, studies have shown that Aβ fibrillization is accelerated and binding is enhanced when incubated with artificially engineered phospholipid vesicles due to the vesicles’ high membrane curvature (~30 nm diameter; Sugiura et al., 2015). However, it has not been reported whether the extent of the Aβ aggregation observed in this *in vitro* study is comparable to *in vivo* exosomes or SVs.

## EV Biogenesis and Lipid Composition

Intercellular communication by way of small lipid-bound vesicles, known as EVs, are essential and evolutionarily conserved throughout all multicellular organisms (Lawson et al., 2017). EVs are released from many cell types, including neurons, into the extracellular space and carry proteins, lipids, and genetic cargo (Thery et al., 2002; Zhang et al., 2015; Lawson et al., 2017). EVs are composed of a lipid bilayer that not only protects the encapsulated products but also influences extracellular trafficking and membrane fusion to recipient cells (Skotland et al., 2017).There are varying types of EVs, but they can be roughly categorized as either exosomes (50–100 nm in size) or microvesicles (100–1,000 nm in size; Stahl et al., 2019). Of the two subtypes, exosomes and their similar but smaller counterpart (~40 nm), synaptic vesicles (SVs), are the most well studied and are shown to participate in the spread of aggregated proteins involved in neurodegenerative diseases such as AD and PD (Coleman and Hill, 2015; Skotland et al., 2017; Vogel et al., 2020; Gagliardi et al., 2021).

**Figure 1:**
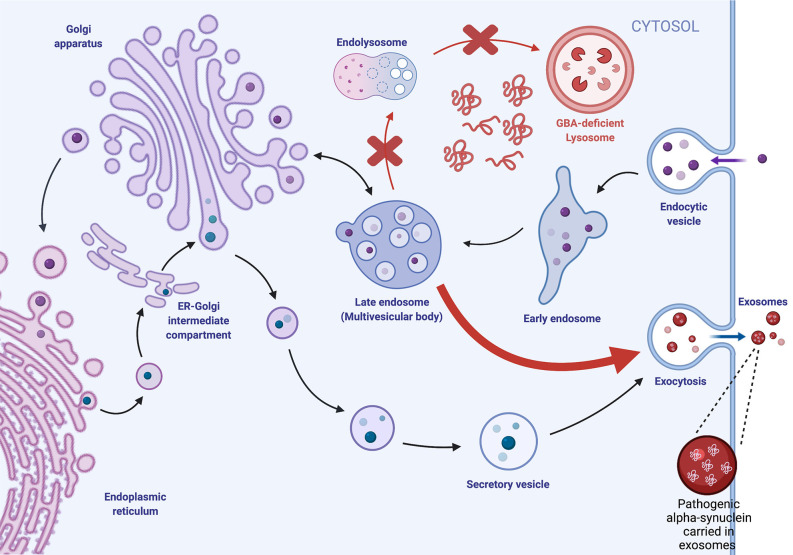
Exosome biogenesis is dysregulated by lysosomal GBA deficiencies. Extracellular proteins are endocytosed and trafficked intracellularly *via* endocytes, where they undergo multiple rounds of inward budding to generate intraluminal vesicles (ILVs) within late endosomes/multivesicular bodies (MVBs). MVBs can fuse with the lysosome for degradation or fuse with the plasma membrane, where ILVs are released as extracellular vesicles (EVs). We hypothesize that GBA deficiency impairs lysosomal degradation, leading to the accumulation of intracellular protein aggregates and increased trafficking of the MVB to the plasma membrane for EV biogenesis.

Lipids are essential for the biogenesis and formation of exosomes as they form the lipid bilayer of the vesicles. Biogenesis of exosomes initiates when the cytoplasmic membrane invaginates extracellular proteins and buds inward to generate early endosomes. Early endosomes then go through another round of inward budding, leading to the formation of intraluminal vesicles (ILVs) and late endosomes. Late endosomes encompassing ILVs are recognized as multivesicular bodies (MVBs). The MVBs either fuse with lysosomes for lysosomal degradation or fuse with the plasma membrane for exocytosis generation of exosomes (Figure 1). Canonical endolysosomal trafficking is mediated by Endosomal Sorting Complexes Required for Transport (ESCRT) complexes and small Rab GTPases (Weeratunga et al., 2020). Regulation of how ESCRT machinery sorts endocytic cargo for recycling and degradation continues to be an active area of discovery and has revealed lipid mediators such as phosphoinositides and lipid transporters (Shen et al., 2011; Stalder and Gershlick, 2020). In addition to the ESCRT-mediated exosome biogenesis pathway, there is also an ESCRT-independent biogenesis pathway that relies on neutral sphingomyelinase (nSMase; Trajkovic et al., 2008; Menck et al., 2017). Neutral sphingomyelinase hydrolyzes membrane lipid sphingomyelin, generating ceramide and phosphocholine. Sphingomyelin hydrolysis is also an important pathway for larger microparticle EV biogenesis in both astrocytes and glia (Bianco et al., 2009).

**Figure 2:**
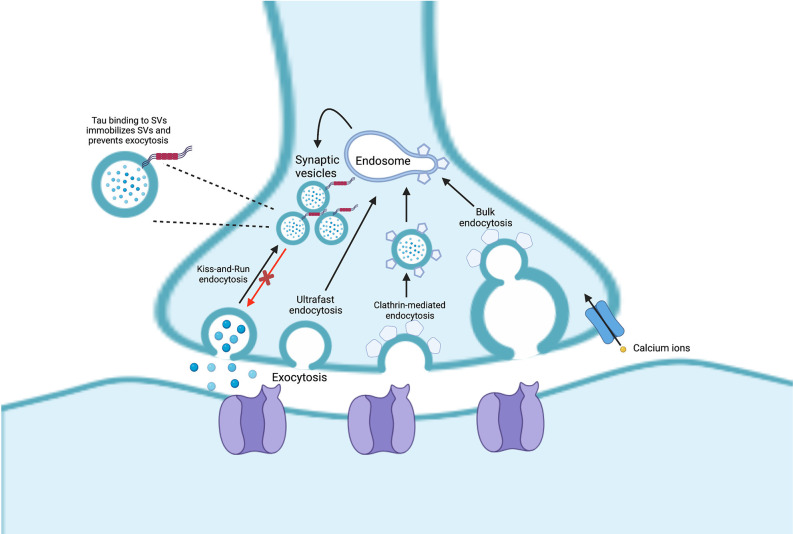
Endocytosis of synaptic vesicles (SVs) can occur through four pathways: (1) “kiss-and-run” endocytosis; (2) ultrafast endocytosis; (3) clathrin-mediated endocytosis; and (4) bulk endocytosis. In Alzheimer’s disease (AD), pathogenic tau can bind to the SVs which can lead them to be less mobile, inducing SV clustering and reducing neurotransmission.

SVs also use endosomal intermediates but differ from exosomes in that fusion with the presynaptic plasma membrane for neurotransmitter release relies on calcium-triggered fusion mediated by synapse-specific machinery (Sudhof and Rizo, 2011; Wu et al., 2014). Endocytosis of SVs can occur through four suggested pathways: (1) “kiss-and-run" endocytosis; (2) clathrin-mediated endocytosis; (3) ultrafast endocytosis; and (4) bulk endocytosis. These endocytic pathways are reviewed in detail in Janas et al. (2016; Figure 2).

Exosomes and SVs have many overlapping features, but also have distinctive protein markers, size, and lipid compositions. Both types of vesicles have been shown to aid the pathogenic spread of neurodegenerative disorders *via* the endocytic pathway, which is where these vesicles are formed. Both exosomes and synaptic vesicles contain similar cholesterol levels (44% mol) that help maintain the arrangement of phospholipids in the membrane’s bilayer and thereby help maintain the rigidity of the vesicle’s membranes (Wang et al., 2020). While both exosomes and synaptic vesicles are equally highly enriched in cholesterol, exosomes are also highly enriched in sphingolipids in the form of sphingomyelin (SM), which is crucial for the structure and rigidity of exosomes. SM d18:1/16:0, SMd18:1/24:0, and SMd18:1/24:1 constitute approximately 35%, 20%, and 20%, respectively of the total SM species in exosomes and play a pivotal role in exosome biogenesis by promoting the budding of exosomes (Skotland et al., 2017). On the other hand, synaptic vesicle membranes have comparatively higher levels of phosphatidylcholine, phosphatidylethanolamine, and phosphatidylinositols (Janas et al., 2016).

## EVs as A Vehicle Mediating Neurodegenerative Disease Progression

Recent studies increasingly suggest that EVs play a critical role in the progression of neurodegenerative diseases. There is mounting evidence supporting upregulation of exosome biogenesis as an alternative strategy for cells when the lysosomal function is impaired. Although upregulation of exosome biogenesis may be a mechanism for the cell to get rid of unwanted proteins, these exosomes appear to also be vehicles for propagating pathogenic protein aggregation, ultimately promoting disease progression.

Aβ fragments and tau have been shown to be secreted through exosomes, which mediate cell to cell transfer of these proteins (Saman et al., 2012; Asai et al., 2015; Wang et al., 2017; Laulagnier et al., 2018; Sardar Sinha et al., 2018). In N2a neuroblastoma cells expressing the APP Swedish mutation, C-terminal fragments of APP resulting from cleavage by β- and gamma-secretases were enriched within secreted exosomes and selectively internalized by recipient neurons (Laulagnier et al., 2018). Exosomes isolated from human AD brain tissues were internalized by co-cultured neuroblastoma cells, resulting in increased cytotoxicity compared to exosomes from control brain tissue (Sardar Sinha et al., 2018). Furthermore, inhibiting exosome formation by knocking down ESCRT proteins TSG101 and VPS4A, which are essential for exosome formation, decreased the number of secreted exosomes and inhibited the spread of Aβ (Sardar Sinha et al., 2018). An ESCRT-independent exosome biogenesis pathway has also been identified, which relies on neutral sphingomyelinase (nSMase; Menck et al., 2017). Neutral sphingomyelinase hydrolyzes membrane lipid sphingomyelin, generating ceramide. This lipid-dependent exosome pathway is relevant to neurodegeneration, as inhibition of neutral sphingomyelinase 2 (nSMase2) with the inhibitor GW4869 in 5XFAD mice significantly reduced exosome production, brain ceramide levels, and Aβ plaque pathology (Dinkins et al., 2016). Together, these studies support a role for exosomes in propagating AD pathology.

Exosomes are also implicated in the propagation of Lewy pathology in PD. α-syn is normally membrane-bound and associated with synaptic vesicles. In disease contexts, it has been observed to be packaged both within exosomes and associated with the outer membrane of exosomes. Neuroblastoma SH-SY5Y cells expressing wildtype α-syn were found to secrete α-syn monomers and oligomers extracellularly in association with exosomes in a calcium-dependent manner (Emmanouilidou et al., 2010) as well as when the lysosomal function was inhibited (Alvarez-Erviti et al., 2011). Several subsequent studies have confirmed the association of α-syn with secreted EVs, reviewed in Emmanouilidou and Vekrellis (2016). Internalization of α-syn-containing exosomes by normal SH-SY5Y cells resulted in increased α-syn within the recipient cells, demonstrating that exosomes can be a vehicle for transferring and propagating pathogenic proteins to other cells (Emmanouilidou et al., 2010; Alvarez-Erviti et al., 2011). Similar findings were observed in human neuroglioma and rat primary cortical neuron cultures overexpressing human wild-type α-syn, where inhibiting autophagy increased production of EVs containing α-syn as well as proteins associated with autophagy such as p62, LC3II, and LAMP2A (Minakaki et al., 2018). Exosomes isolated from CSF of PD patients and injected into a reporter cell line for α-syn oligomerization resulted in increased oligomerization (Stuendl et al., 2016). Subsequent studies have replicated these findings *in vivo*, where exosomes isolated from CSF or brain tissue of PD and DLB patients were injected into mouse brains, resulting in α-syn oligomerization around the site of injection of EVs (Ngolab et al., 2017; Minakaki et al., 2018). These studies further indicate that EVs can mediate cell to cell transfer of α-syn pathology.

*GBA* mutations appear to have a similar effect in impairing lysosome function, resulting in increased production of EVs carrying pathogenic proteins such as α-syn that propagate pathogenic aggregates in recipient cells. Our work using a *Drosophila* model of GBA deficiency not only revealed increased protein aggregation but also upregulation of proteins associated with EV biogenesis and dysregulation of EVs, including increased levels of EV-intrinsic proteins and Ref(2)p, the *Drosophila* homolog of p62 (Davis et al., 2016c; Thomas et al., 2018). A similar finding was observed in *synuclein* A53T mice treated with Conduritol B epoxide (CBE) to inhibit GCase enzyme activity, resulting in an increase in α-syn-containing exosomes (Papadopoulos et al., 2018). Furthermore, tissue-specific restoration of wildtype *GBA* in *GBA* mutant flies revealed non-cell autonomous rescue of protein aggregation in distant tissues including the brain (Jewett et al., 2021). This non-cell autonomous suppression of protein aggregation was accompanied by normalization of the dysregulation in EVs observed in *GBA* mutant flies (Jewett et al., 2021). Together, these results suggest that GBA deficiency accelerates PD pathogenesis through the propagation of protein aggregates by dysregulating exosome biogenesis. This mechanism could support why *GBA* mutations are observed to associate with faster progression of PD, as the upregulation in exosome biogenesis may accelerate the spread of Lewy pathology.

Synapses are crucial sites for chemical communication between neurons and play a vital role in the dynamic functions of the brain. *In vivo* studies in mice and humans indicate that tau protein aggregation spreads readily to trans-synaptic regions of the brain, although exosomes at the synaptic cleft, not synaptic vesicles, may be mediating propagation of tau aggregates (Liu et al., 2012; de Calignon et al., 2012; Vogel et al., 2020; Miyoshi et al., 2021). In AD, synaptic dysfunction is considered to be an early pathological event, preceding Aβ deposits in the brain (Marsh and Alifragis, 2018). Though accumulation of hyperphosphorylated tau in NFTs is a hallmark of AD, soluble, non-aggregated forms of tau appear to be the main toxic element inducing early synaptic deficits (Ahmed et al., 2014; Zhou et al., 2017). In studies using fly and rodent models and post-mortem human brain, tau has been shown to associate with SVs either through protein binding or through direct membrane interactions (Zhou et al., 2017). Tau binding to SVs leads them to be crosslinked and less mobile, thereby inducing SV clustering and attenuating neurotransmission (McInnes et al., 2018).

## Lipid Alterations Influencing Exosomes and Progression of Neurodegeneration

Lipid metabolism alterations influence multiple aspects of EV biogenesis, including the vesicular trafficking steps leading to exosome release, exosome morphology, and the selective cargo associated with EVs. Lipid composition strongly dictates the curvature of the lipid membrane, and because EVs are so small, the surface area and curvature of the inner membrane can differ significantly from the outer membrane (Skotland et al., 2017). EV membranes are enriched in lipids with monounsaturated fatty acyl groups compared to plasma membranes, likely due in part to the high curvature of EV membranes (Skotland et al., 2017). Unsaturation of fatty acyl chains of ceramides allows for denser packing of ceramide molecules and reduced fluidity of membranes. The length of fatty acyl chains can also influence membrane curvature, with shorter fatty acyl chains positively affecting the curvature in lipid monolayers (Skotland et al., 2017).

Alterations in lipid density and membrane curvature led to various α-syn structural changes and alterations in binding affinity, suggesting that the curvature of the membrane may be a key influence in driving interactions promoting α-syn oligomerization (Middleton and Rhoades, 2010; Pranke et al., 2011). The binding of α-syn to negatively charged lipids within the membrane was also observed to induce conformational changes of EV membranes, increasing vesicle permeability (Hannestad et al., 2020). These studies suggest a bidirectional interaction between the exosome membrane and α-syn that may further enhance neurotoxic effects.

The lipid composition of exosomes also modulates α-syn aggregation, exosome release, and cargo sorting. α-syn has increased affinity to anionic lipids and membranes with increased fluidity (Kjaer et al., 2009; Galvagnion et al., 2016). Systematic analyses of alterations in membrane composition of spherical unilamellar vesicles on *in vitro* α-syn aggregation revealed that gangliosides GM1 and GM3 enrichment accelerated the aggregation of α-syn (Grey et al., 2015), indicating that lipid composition can have a significant influence on α-syn pathology. The reduction of ceramide, which is critical for ESCRT-independent exosome release, with inhibition of neutral sphingomyelinase 2 (nSMase2) reduced the spread of α-syn among co-cultured SH-SY5Y cells (Sackmann et al., 2019). A recent study of brain tissue and CSF of patients with PD or DLB with and without *GBA* mutations found that there was a significant increase in several ceramide species in brain tissue and EVs isolated from CSF from all disease patients compared to control, independent of *GBA* genotype (Kurzawa-Akanbi et al., 2021). Interestingly, proteomic analysis revealed the presence of α-syn, β-syn, gamma-syn, and tau in both disease and control EVs, with no significant difference in levels between either group. However, wild-type α-syn monomers were observed to bind much more readily to disease EVs than control EVs, and this interaction resulted in fibrillization of α-syn, which was absent in the presence of control EVs (Kurzawa-Akanbi et al., 2021). Although it was surprising that the presence of disease rather than *GBA* genotype was associated with α-syn aggregation, lipid alterations, and α-syn-EV interactions, this study interrogated post-mortem tissues from presumably end-stage disease, where the effect of GBA genotype may not be as strong as early and mid-stage disease. These studies suggest that the lipid composition of EVs may be more influential in the progression of neurodegeneration than the EV cargo itself.

In AD, Aβ associates with exosomes as well, although there is conflicting evidence on whether this association promotes or slows the progression of disease (Rajendran et al., 2006). A reduction in the expression of TSG101 and RAB35 and exosome production was observed in a mouse model ApoE4, suggesting that ApoE4 disrupts exosome biogenesis, leading to endolysosomal deficits and Aβ accumulation (Peng et al., 2019). Exosomes were also reduced in human postmortem brain samples of ApoE4 carriers compared to *ApoE3* carriers (Peng et al., 2019). However, the loss of nSMase2 in 5XFAD mice, a model of Aβ aggregation, resulted in decreased ceramide levels and brain exosomes, reducing plaque burden and tau phosphorylation as well as improving cognition on a fear-based conditioning task (Dinkins et al., 2016). It is possible that while overall production of exosomes may be decreased in AD, a subpopulation of exosomes dependent on ceramide metabolism for biogenesis is responsible for the propagation of AD pathology, and further reduction of ceramide levels and exosome production may be neuroprotective. In addition, alterations in lipid composition of EV membranes may have cell-specific effects, as an increase in gangliosides in exosomes from neuronal cells deficient in sphingomyelin synthase 2 (SMS2) resulted in increased uptake and lysosomal degradation of Aβ into co-cultured microglia (Yuyama et al., 2012).

## Glial Role in Propagation of Neurodegenerative Pathology

Much of the focus thus far in understanding the propagation of neurodegenerative protein aggregate pathology has been on neuron-to-neuron transfer. However, the field is beginning to turn its attention to glial cells directly interacting with neurons. Increasing evidence shows that both astrocytes and microglia can uptake neuronal exosomes as well as release exosomes containing neurodegenerative-related proteins and lipids. We will first assess evidence for exosome uptake by glia, which are the most abundant cell type within the brain. We will then review evidence for whether recipient non-neuronal cells are playing a neuroprotective or toxic role to neighboring neurons in their ability to process and propagate aggregate-prone proteins.

Astrocytes are the most abundant glial cell type in the CNS and are essential in supporting and maintaining a homeostatic environment for neurons *via* mechanisms such as glutamate synthesis, vasomodulation, and glycogen storage and metabolism (Phelps, 1972; Stobart and Anderson, 2013; Nortley and Attwell, 2017; Barber and Raben, 2019). Astrocytes support neuronal lipid metabolism by synthesizing lipids associated with synaptic vesicle formation and engulfing oxidized lipids from highly metabolic neurons (Barber and Raben, 2019). Astrocytes also synthesize lipoproteins that mediate cholesterol efflux by neurons and are a reservoir for fatty acids and glycogen that are metabolized by neurons (Gu et al., 2010; Chen et al., 2013). Astrocytes have also been shown to endocytose fatty acids from hyperactive neurons and accumulate lipid droplets, along with shuttling fatty acids to the mitochondria for oxidative phosphorylation and performing lipolysis, thereby preventing neuronal lipotoxicity (Qi et al., 2021).

More recently, astrocytes have been implicated in the propagation of pathogenic protein aggregates. While astrocytes do not express α-syn they have been observed to uptake cytoplasmic pre-aggregate species and oligomeric fibrils of α-syn more readily than neurons (Lee et al., 2010; Gustafsson et al., 2017; Loria et al., 2017; di Domenico et al., 2019; Tsunemi et al., 2020) They have also been shown to uptake Aβ proto-fibrils and aggregates (Mulder et al., 2014; Sollvander et al., 2016; Nilson et al., 2017) and tau monomeric species, pre–formed tau fibrils, and phosphorylated tau aggregates (Martini-Stoica et al., 2018; Perea et al., 2019; Mate De Gerando et al., 2021). Furthermore, astrocytes uptake Aβ more readily when packaged within EVs (Dinkins et al., 2016), as well as α-syn associated with EVs (Tsunemi et al., 2020). In post-mortem AD brains, astrocytes were found to localize around amyloid-plaques and hyperphosphorylated tau in hippocampal regions (Richetin et al., 2020). Similar findings were observed in an AD mouse model, where astrocytes surrounded Aβ aggregates (Dinkins et al., 2016). This evidence suggests a protective role of astrocytes where in addition to internalizing free extracellular pathogenic proteins associated with neurodegeneration, astrocytes can also endocytose pathogenic proteins associated with EVs, and may do this more efficiently than neurons. These studies have led to the hypothesis that astrocytic uptake of secreted extracellular aggregate-prone proteins assists in preventing the uptake of proteins that otherwise would be endocytosed by neurons and seed further neuronal spread of pathology.

Astrocytes’ role in neurodegenerative contexts is further assessed by studies examining neurodegenerative genetic perturbations. Expressing α-syn with the familial PD mutation A53T in astrocytes of mice caused widespread astrogliosis in multiple brain regions and increased levels of α-syn aggregation (Gu et al., 2010). Dysregulation of the blood-brain barrier, as evidenced by altered Aquaporin 4 and Glut1 expression, and reduced expression of excitatory amino acid transporters was also observed, suggesting impairment in astrocytic function in maintaining neuronal excitation homeostasis (Gu et al., 2010). Astrocytes derived from iPSCs from patients with *ATP13A2* mutations causing familial parkinsonism had reduced endocytosis of neuronal EV-associated α-syn (Tsunemi et al., 2020). When *ATP13A2* mutant astrocytes were co-cultured with control neurons, there was an increase in neuronal intracellular α-syn, indicating that astrocytes normally have a neuroprotective role in reducing neuronal pathology.

**Figure 3:**
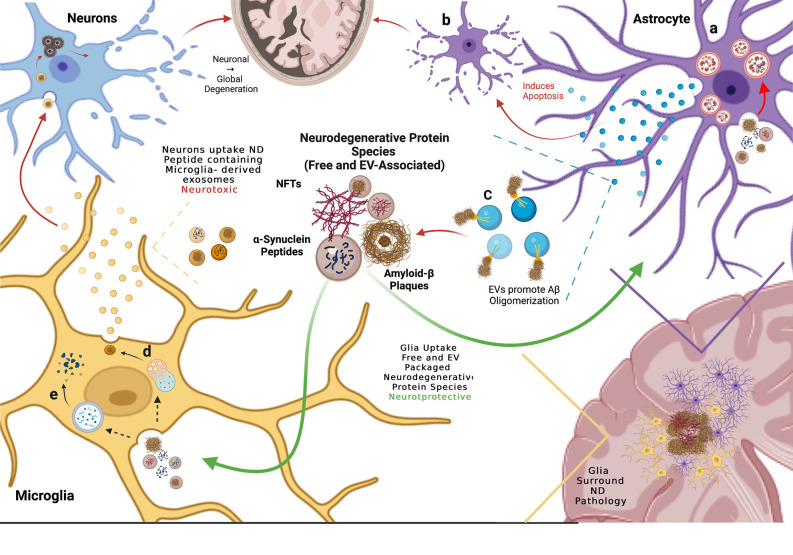
Glial cells mediate propagation of neurodegenerative protein species by up-taking both free and extra-cellular vesicle (EV)-associated proteins, thereby preventing neuronal uptake and spread of disease. **(A)** Astrocytes endocytose free and EV-associated aggregate prone proteins including α-syn and Aβ and process them in the lysosome but subsequent lysosomal dysfunction results in debris-burdened enlarged lysosomes that do not degrade the engulfed material. **(B)** When exposed to Aβ, astrocytes release EVs that induce apoptosis in neighboring astrocytes. **(C)** Astrocytes also release EVs enriched in glycosphingolipids (GSLs) that bind monomeric Aβ, promoting oligomerization and plaque formation. **(D)** Microglia uptake both free and EV-associated aggregate-prone proteins and process them through the endolysosomal system where they are repackaged into EVs that are released and engulfed by neighboring neurons, presenting evidence of neurotoxic propagation from glia to neurons. **(E)** Microglia degrade endocytosed pathogenic proteins *via* phagocytosis, clearing aggregate prone proteins.

However, once endocytosed, astrocytes may not effectively degrade aggregate-prone proteins, leading to increased neurotoxic effects. In iPSC-derived astrocytes co-cultured with neurons, astrocytes showed preferential endocytosis of neuronal secreted α-syn, but α-syn subsequently accumulated within astrocytes and co-localized with LAMP-2 over several days, suggesting that lysosomal degradation was impaired (di Domenico et al., 2019; Figure 3A). Similarly, when primary mouse neurons, astrocytes, and oligodendrocytes were co-cultured and exposed to recombinant labeled α-syn oligomers, astrocytes rapidly took up most of the α-syn oligomers compared to neurons and oligodendrocytes (Lindstrom et al., 2017). However, α-syn oligomers remained detectable and co-localized with LAMP-1 for over 12 days after uptake, and astrocytes developed evidence of mitochondrial damage (Lindstrom et al., 2017). In addition, death of co-cultured neurons was observed 12 days after exposure to the α-syn oligomers, coinciding with the impairments in endolysosomal trafficking and mitochondrial defects in the astrocytes, suggesting that astrocytes may be producing non-cell autonomous neurotoxic signals in response to the endocytosed α-syn oligomers. Rat primary astrocytes were also observed to uptake α-syn oligomers, this time purified from postmortem brain tissue of PD patients, more efficiently than rat primary cortical neurons (Cavaliere et al., 2017). Cavaliere et al. (2017) cultured neurons and astrocytes in a microfluidic chamber system to test whether α-syn oligomers exposed to neurons or astrocytes could be transferred to neurons or astrocytes. Consistent with other studies, they found that α-syn oligomers endocytosed by astrocytes led to increased neuronal cell death compared to direct uptake of α-syn oligomers by neurons. These studies suggest that endocytosis of α-syn may cause astrocytes to become neurotoxic reactive astrocytes.

Astrocytes can also uptake tau pathology, which can be enhanced by lysosomal function. Mate De Gerando et al. (2021) confirmed that tau can be transferred between neurons and astrocytes *in vivo* in a mouse model by expressing a pro-aggregating tau peptide in neurons and human wild-type tau in astrocytes in the hippocampus. Using an antibody that only detects the aggregates formed by co-expression of both forms of tau, positive tau-aggregate staining was observed in both cell types, indicating transfer of tau species between neurons and astrocytes. Martini-Stoica et al. (2018) overexpressed Transcription Factor EB (TEFB), a master regulator of lysosomal biogenesis, in astrocytes and found that this not only increased trafficking of pre-formed fibrillary tau fibrils to the lysosome but also increased uptake of these fibrils. Increased expression of TFEB in astrocytes in the PS19 tauopathy mouse model reduced tau pathology and decreased levels of astrocyte gliosis, although increased expression of astrocytic TFEB in a more rapidly progressive tauopathy mouse model did not reduce tau pathology. These studies suggest that healthy astrocytes can process tau oligomers effectively *via* endolysosomal trafficking to reduce the spread of tau pathology and gliosis.

However, astrocytes may also release exosomes that mediate the spread of pathogenic protein aggregates. Astrocytes localized near Aβ peptides and aggregates in whole mouse brains upregulated ceramide production, resulting in increased biogenesis of ceramide (specifically C18:0, C24:0)-enriched exosomes that induced an apoptotic response in neighboring astrocytes (Wang et al., 2012; Dinkins et al., 2016; Figure 3B). The astrocyte-derived exosomes released in response to Aβ exposure also contained phosphorylated-tau (p-tau), a precursor for neurofibrillary tau tangles (NFTs; Chiarini et al., 2017). This suggests that astrocytes recycle endocytosed pathogenic cargo, re-releasing them as astrocytic EVs and potentially facilitating the spread of disease.

Astrocyte-derived exosomes may have distinctive lipid composition compared to neuronal-derived exosomes which could affect protein aggregation and internalization by recipient cells. In addition to enrichment in ceramides (Wang et al., 2012), the ganglioside GM1 was found in the outer layer of exosomes released by astrocytes in the aggressive Alzheimer’s mouse 5XFAD model. Of note, GSLs are more abundant in neuronal exosomes relative to glial exosomes, which have been implicated in the binding of monomeric Aβ (Yuyama et al., 2014). Exosomes isolated from primary astrocytes were observed to induce aggregation of monomeric Aβ_1–42_ peptide 20-fold greater than amyloid alone, suggesting that astrocytic exosomes facilitate the formation of Aβ plaques within the AD brain (Dinkins et al., 2014). Adding an anti-ceramide antibody reduced Aβ aggregation, suggesting that the ceramide enrichment of astrocyte-derived exosomes is required for monomeric Aβ binding and oligomerization (Dinkins et al., 2016). Therefore, the efficacy of astrocyte-derived exosomes as a vehicle for Aβ plaque formation and spread appears to be dependent on lipid composition.

In conclusion, evidence suggests that astrocytes reduce the neuronal spread of pathology by readily taking up free extracellular or EV-associated neurodegenerative proteins due to more efficient endolysosomal trafficking compared to neurons. However, it remains unclear whether astrocytic uptake of neuronal exosomes and pathogenic proteins is ultimately neuroprotective or neurotoxic. These glial cells may eventually accumulate pathogenic proteins, leading to the release of astrocytic exosomes that further propagate pathogenic aggregate pathology and conversion to reactive cytotoxic astrocytes (Figure 3). Further work will need to address the possible neuroprotective vs. neurotoxic roles of astrocytes in the propagation of pathogenic proteins in neurodegeneration.

There is increasing recognition of microglia in the clearance of pathogenic proteins in multiple neurodegenerative conditions. Microglia are the primary immune cells of the CNS that become active under conditions of injury, infection, or even neurodegeneration. When active they are phagocytic, assuming a macrophage-like phenotype where they become mobile and endocytose surrounding debris (Neumann et al., 2009). They have been observed to migrate to newly formed amyloid plaques within 24 h of formation in an aggressive AD mouse model and prevent the continued formation of those plaques (Meyer-Luehmann et al., 2008).

Recruitment of microglia to Aβ plaques followed by clearance of Aβ was also observed in the transgenic mouse model PDAPP in which APP, the pre-aggregate form of Aβ, is over expressed. In 20-month-old mice corteces, an anti-Aβ antibody 10D5 was injected at sites of already existing Aβ plaques in an attempt to see if this would promote the clearance of the plaques. Microglia were recruited to the site of injection in high abundance 3 days post-injection, coinciding with clearance of the anti-Aβ-stained plaques. Though this clearance was mediated by immunotherapy, it is still positive evidence for microglia to surround and clear Aβ and associated bodies, thereby preventing the spread of Aβ plaques (Bacskai et al., 2001). Microglial internalized Aβ was degraded *via* autophagy as confirmed with co-staining of LAMP1, CD68 and Aβ (Bacskai et al., 2001; Feng et al., 2020). These studies support a neuroprotective role for microglia in clearing and halting the propagation of pathogenic senile plaques. Microglia are also recruited to NFTs and internalize insoluble and soluble tau species in AD postmortem tissue, suggesting that microglia continually surveil for aberrant tau (Bolos et al., 2016; Nilson et al., 2017). In an AD mouse model, microglia have been observed to localize around phosphorylated tau within the dentate gyrus and phagocytose p-tau positive neurons, preventing the spread of phosphorylated tau and progression of neurodegeneration (Asai et al., 2015; Loria et al., 2017).

Microglia are also able to phagocytose exosomes containing Aβ, tau, and α-syn (Yuyama et al., 2012; Wang et al., 2017; Xia et al., 2019). However, microglial phagocytosis may not be neuroprotective, as internalized proteins are recycled and released back into the extracellular matrix as microglial exosomes, where they can further propagate neurodegeneration (Asai et al., 2015; Guo et al., 2020). Depletion of microglia in a mouse AD model resulted in decreased EV-associated oligomeric tau and reduced tau pathology in the brain. The same findings were observed when exosome production was inhibited in microglia *via* the nSMase ceramide synthesis pathway (Asai et al., 2015). Similar proof of microglia-derived exosome mediating propagation has been observed with α-syn, where microglial exosomes containing α-syn were internalized by co-cultured neurons and led to increased α-syn oligomerization . In the same co-culture experiment, when microglial nSMase was inhibited to impair exosome biogenesis, oligomerization of α-syn in neurons decreased (Guo et al., 2020) Thus, evidence supports both a neuroprotective role for microglia in internalizing pathogenic proteins neurons, as well as promoting the propagation of protein aggregation in neurodegeneration.

Membrane lipid composition of EVs also influences microglial exosome internalization. Yuyama et al. observed that GSLs on the outer membrane of exosomes were essential for Aβ binding and oligomerization. However, there was no effect on microglial internalization of glycosphingolipid-depleted exosomes. Previously, this group showed that microglia require phosphatidylserine (PS), a lipid implicated in the autophagy of cellular debris and expressed in the outer membrane of apoptotic cells, was required for microglia to uptake neuronal exosomes (Neumann et al., 2009; Yuyama et al., 2012, 2014). It should be noted that another glial cell type, oligodendrocytes which provide the myelin sheath surrounding neuronal axons in the CNS, have been observed to accumulate tau aggregates (Narasimhan et al., 2020) and α-syn filaments (Tu et al., 1998). Accumulation of these pathogenic aggregates led to oligodendrocyte cell death and neurotoxicity (Tu et al., 1998; Narasimhan et al., 2020), adding further complexity to the non-cell autonomous interactions contributing to the spread of pathogenic protein aggregation and progression of neurodegeneration.

In addition to proteins and lipids, EVs contain nucleic acids including microRNAs (miRNAs) that may mediate interactions with neurons. Analysis of EVs from primary rat microglia cultured with inflammatory Th1 cytokines revealed a microglial-specific miRNA that reduces dendritic spine density when internalized by hippocampal neurons (Prada et al., 2018). Specific extracellular and EV-associated miRNAs in CSF have been found to be associated with neurodegenerative diseases such as AD and PD (Lusardi et al., 2017; Grossi et al., 2021). Characterization of miRNAs trafficked to EVs and whether they mediate neuroprotective or neurotoxic effects in a neurodegenerative context is an emerging area of study.

## Conclusion

The spread of pathologic protein aggregates throughout regions of the brain in neurodegenerative diseases is associated with the progression of clinical disease. A significant amount of evidence now supports EVs as important vehicles mediating the propagation of pathogenic protein aggregates in multiple neurodegenerative diseases. Alterations in lipid metabolism also alter disease progression by promoting oligomerization of aggregate-prone pathogenic proteins and impairing EV biogenesis. Although this review focused on the neuropathology related to AD and PD, EVs and lipid metabolism alterations are implicated in several other neurodegenerative diseases characterized by pathogenic protein aggregates, including DLB, Huntington’s, ALS, prion disease, and other tauopathies and α-synucleinopathies. While the specific proteins involved in aggregates for each of these diseases are unique, there may be a common underlying pathogenic mechanism regulating the spread of these aggregates that could be exploited therapeutically to slow or halt neurodegeneration. Elucidating the alterations in lipid metabolism may reveal possible druggable targets in lipid pathways to ameliorate neurodegenerative processes, as well as identify potential biomarkers of disease progression.

Studies of tissues from *GBA* carriers and GBA deficient model organisms and cultured cells have provided some of the most compelling evidence for the role of lipid metabolism in the progression of neurodegeneration. Lipid composition has widespread cellular influences affecting disease progression, including cell autonomous vesicular trafficking and autophagy, exosome biogenesis, cell signaling, and cell survival. In this review, we examined the evidence for the lipid composition of membranes directly interacting with neurodegenerative proteins to promote aggregation and pathology. Ceramide metabolism is of particular interest, given its multiple avenues of influences that are all implicated in neurodegeneration, including exosome biogenesis, membrane fluidity, and curvature, cell signaling, regulation of apoptosis, autophagy, and inflammation. These studies suggest that alterations in lipid metabolism can have a profound effect in accelerating disease progression. As the lipid abnormalities found in AD, PD and other aggregate-prone neurodegenerative diseases have similarities in common in modifying protein aggregation, elucidation of lipid-mediated mechanisms to slow neurodegeneration holds significant promise as a therapeutic target. Improvements in detection and annotation of lipids from tissues of patients, and animal or cell culture models are resulting in a complex lipid landscape that is increasingly difficult to interpret. Continued lipidomic analysis of patient tissues, isogenically controlled model organisms, and cell culture models will be crucial to identify the key lipid alterations involved in pathogenesis and disease progression.

Understanding the role of glia in the propagation of protein aggregation and disease progression is becoming a major focus in neurodegeneration. However, it remains unclear which glial cells may have neuroprotective vs. neurotoxic effects. Several studies suggest that glia may initially have neuroprotective effects in clearing extracellular pathogenic free-floating and EV-associated proteins that would otherwise seed and propagate pathology in recipient neurons. However, uptake of pathogenic EVs may overwhelm glial endolysosomal trafficking, leading to toxicity of glia as further propagation of pathologic proteins within glial EVs. Recent studies using co-cultures of iPSC-derived neurons and glia are starting to elucidate the complex interactions between these cell types in health and disease. Investigations using iPSC-derived organoid models may be necessary to further understand complex interactions between these cell types that are perturbed in neurodegenerative diseases. Further studies are necessary to elucidate the role of glia in reducing or promoting the propagation of neurodegenerative pathology. These studies could reveal alternative therapeutic targets in harnessing the increased uptake of pathogenic aggregate-prone proteins by glia to reduce the spread of neurodegenerative pathology and slow disease progression.

Lipid metabolism alterations contribute to the progression of neurodegeneration through numerous pathways influencing the spread of pathology. While much focus has been on the study of these aggregate-prone proteins, the lipids surrounding these proteins in EVs, endocytic vesicles, lipid rafts, and cell membranes are altered in the context of neurodegeneration, promoting aggregation and influencing the spread of pathogenic protein aggregates from cell to cell, suggesting that lipid alterations may be as important as the pathogenic proteins themselves.

## Author Contributions

REE, BL, AK, and MYD performed the literature review and wrote the manuscript. All authors contributed to the article and approved the submitted version.

## Conflict of Interest

The authors declare that the research was conducted in the absence of any commercial or financial relationships that could be construed as a potential conflict of interest.

## Publisher’s Note

All claims expressed in this article are solely those of the authors and do not necessarily represent those of their affiliated organizations, or those of the publisher, the editors and the reviewers. Any product that may be evaluated in this article, or claim that may be made by its manufacturer, is not guaranteed or endorsed by the publisher.

## References

[B1] Abed Rabbo M. Khodour Y. Kaguni L. S. Stiban J. (2021). Sphingolipid lysosomal storage diseases: from bench to bedside. Lipids Health Dis. 20:44. 10.1186/s12944-021-01466-0 33941173 PMC8094529

[B2] Ahmed Z. Cooper J. Murray T. K. Garn K. Mcnaughton E. Clarke H.. (2014). A novel *in vivo* model of tau propagation with rapid and progressive neurofibrillary tangle pathology: the pattern of spread is determined by connectivity, not proximity. Acta Neuropathol. 127, 667–683. 10.1007/s00401-014-1254-6 24531916 PMC4252866

[B3] Alcalay R. N. Hsieh F. Tengstrand E. Padmanabhan S. Baptista M. Kehoe C.. (2020). Higher urine bis(Monoacylglycerol)phosphate levels in LRRK2 G2019S mutation carriers: implications for therapeutic development. Mov. Disord. 35, 134–141. 10.1002/mds.27818 31505072 PMC6981003

[B4] Alcalay R. N. Levy O. A. Waters C. C. Fahn S. Ford B. Kuo S. H.. (2015). Glucocerebrosidase activity in Parkinson’s disease with and without GBA mutations. Brain 138, 2648–2658. 10.1093/brain/awv179 26117366 PMC4564023

[B5] Alecu I. Bennett S. A. L. (2019). Dysregulated lipid metabolism and its role in alpha-synucleinopathy in Parkinson’s disease. Front. Neurosci. 13:328. 10.3389/fnins.2019.00328 31031582 PMC6470291

[B6] Alessenko A. V. Bugrova A. E. Dudnik L. B. (2004). Connection of lipid peroxide oxidation with the sphingomyelin pathway in the development of Alzheimer’s disease. Biochem. Soc. Trans. 32, 144–146. 10.1042/bst0320144 14748735

[B7] Alvarez-Erviti L. Seow Y. Schapira A. H. Gardiner C. Sargent I. L. Wood M. J.. (2011). Lysosomal dysfunction increases exosome-mediated alpha-synuclein release and transmission. Neurobiol. Dis. 42, 360–367. 10.1016/j.nbd.2011.01.029 21303699 PMC3107939

[B8] Asai H. Ikezu S. Tsunoda S. Medalla M. Luebke J. Haydar T.. (2015). Depletion of microglia and inhibition of exosome synthesis halt tau propagation. Nat. Neurosci. 18, 1584–1593. 10.1038/nn.4132 26436904 PMC4694577

[B9] Bacskai B. J. Kajdasz S. T. Christie R. H. Carter C. Games D. Seubert P.. (2001). Imaging of amyloid-beta deposits in brains of living mice permits direct observation of clearance of plaques with immunotherapy. Nat. Med. 7, 369–372. 10.1038/85525 11231639

[B10] Bancher C. Braak H. Fischer P. Jellinger K. A. (1993). Neuropathological staging of Alzheimer lesions and intellectual status in Alzheimer’s and Parkinson’s disease patients. Neurosci. Lett. 162, 179–182. 10.1016/0304-3940(93)90590-h 8121624

[B11] Barber C. N. Raben D. M. (2019). Lipid metabolism crosstalk in the brain: glia and neurons. Front. Cell. Neurosci. 13:212. 10.3389/fncel.2019.00212 31164804 PMC6536584

[B12] Bianco F. Perrotta C. Novellino L. Francolini M. Riganti L. Menna E.. (2009). Acid sphingomyelinase activity triggers microparticle release from glial cells. EMBO J. 28, 1043–1054. 10.1038/emboj.2009.45 19300439 PMC2664656

[B13] Bolos M. Llorens-Martin M. Jurado-Arjona J. Hernandez F. Rabano A. Avila J. (2016). Direct evidence of internalization of tau by microglia *in vitro* and *in vivo*. J. Alzheimers Dis. 50, 77–87. 10.3233/JAD-150704 26638867

[B14] Braak H. Alafuzoff I. Arzberger T. Kretzschmar H. Del Tredici K. (2006). Staging of Alzheimer disease-associated neurofibrillary pathology using paraffin sections and immunocytochemistry. Acta Neuropathol. 112, 389–404. 10.1007/s00401-006-0127-z 16906426 PMC3906709

[B15] Braak H. Braak E. (1991). Neuropathological stageing of Alzheimer-related changes. Acta Neuropathol. 82, 239–259. 10.1007/BF00308809 1759558

[B16] Braak H. Del Tredici K. Rub U. De Vos R. A. Jansen Steur E. N. Braak E. (2003). Staging of brain pathology related to sporadic Parkinson’s disease. Neurobiol. Aging 24, 197–211. 10.1016/s0197-4580(02)00065-9 12498954

[B17] Brockmann K. Srulijes K. Pflederer S. Hauser A. K. Schulte C. Maetzler W.. (2015). GBA-associated Parkinson’s disease: reduced survival and more rapid progression in a prospective longitudinal study. Mov. Disord. 30, 407–411. 10.1002/mds.26071 25448271

[B18] Cavaliere F. Cerf L. Dehay B. Ramos-Gonzalez P. De Giorgi F. Bourdenx M.. (2017). *in vitro* alpha-synuclein neurotoxicity and spreading among neurons and astrocytes using Lewy body extracts from Parkinson disease brains. Neurobiol. Dis. 103, 101–112. 10.1016/j.nbd.2017.04.011 28411117

[B19] Chen J. Zhang X. Kusumo H. Costa L. G. Guizzetti M. (2013). Cholesterol efflux is differentially regulated in neurons and astrocytes: implications for brain cholesterol homeostasis. Biochim. Biophys. Acta 1831, 263–275. 10.1016/j.bbalip.2012.09.007 23010475 PMC3534809

[B20] Chew H. Solomon V. A. Fonteh A. N. (2020). Involvement of lipids in Alzheimer’s disease pathology and potential therapies. Front. Physiol. 11:598. 10.3389/fphys.2020.00598 32581851 PMC7296164

[B400] Chiarini A. Armato U. Gardenal E. Gui L. Dal Prà I. (2017). Amyloid β-exposed human astrocytes overproduce phospho—tau and overrelease it within exosomes, effects suppressed by calcilytic NPS 2143—further implications for Alzheimer’s therapy. Front. Neurosci. 11:217. 10.3389/fnins.2017.00217 28473749 PMC5397492

[B21] Coleman B. M. Hill A. F. (2015). Extracellular vesicles–Their role in the packaging and spread of misfolded proteins associated with neurodegenerative diseases. Semin. Cell Dev. Biol. 40, 89–96. 10.1016/j.semcdb.2015.02.007 25704308

[B22] Czubowicz K. Jesko H. Wencel P. Lukiw W. J. Strosznajder R. P. (2019). The role of ceramide and sphingosine-1-phosphate in Alzheimer’s disease and other neurodegenerative disorders. Mol. Neurobiol. 56, 5436–5455. 10.1007/s12035-018-1448-3 30612333 PMC6614129

[B23] Davis A. A. Andruska K. M. Benitez B. A. Racette B. A. Perlmutter J. S. Cruchaga C. (2016a). Variants in GBA, SNCA and MAPT influence Parkinson disease risk, age at onset and progression. Neurobiol. Aging 37, 209.e1–209.e7. 10.1016/j.neurobiolaging.2015.09.014 26601739 PMC4688052

[B24] Davis M. Y. Johnson C. O. Leverenz J. B. Weintraub D. Trojanowski J. Q. Chen-Plotkin A.. (2016b). Association of GBA mutations and the E326K polymorphism with motor and cognitive progression in parkinson disease. JAMA Neurol. 73, 1217–1224. 10.1001/jamaneurol.2016.2245 27571329 PMC5056861

[B25] Davis M. Y. Trinh K. Thomas R. E. Yu S. Germanos A. A. Whitley B. N.. (2016c). Glucocerebrosidase deficiency in drosophila results in alpha-synuclein-independent protein aggregation and neurodegeneration. PLoS Genet. 12:e1005944. 10.1371/journal.pgen.1005944 27019408 PMC4809718

[B26] de Calignon A. Polydoro M. Suarez-Calvet M. William C. Adamowicz D. H. Kopeikina K. J.. (2012). Propagation of tau pathology in a model of early Alzheimer’s disease. Neuron 73, 685–697. 10.1016/j.neuron.2011.11.033 22365544 PMC3292759

[B27] di Domenico A. Carola G. Calatayud C. Pons-Espinal M. Munoz J. P. Richaud-Patin Y.. (2019). Patient-specific iPSC-derived astrocytes contribute to non-cell-autonomous neurodegeneration in Parkinson’s disease. Stem Cell Rep.. 12, 213–229. 10.1016/j.stemcr.2018.12.011 30639209 PMC6372974

[B28] Dinkins M. B. Dasgupta S. Wang G. Zhu G. Bieberich E. (2014). Exosome reduction *in vivo* is associated with lower amyloid plaque load in the 5XFAD mouse model of Alzheimer’s disease. Neurobiol. Aging 35, 1792–1800. 10.1016/j.neurobiolaging.2014.02.012 24650793 PMC4035236

[B29] Dinkins M. B. Enasko J. Hernandez C. Wang G. Kong J. Helwa I.. (2016). Neutral sphingomyelinase-2 deficiency ameliorates Alzheimer’s disease pathology and improves cognition in the 5XFAD mouse. J. Neurosci. 36, 8653–8667. 10.1523/JNEUROSCI.1429-16.2016 27535912 PMC4987436

[B31] Emmanouilidou E. Melachroinou K. Roumeliotis T. Garbis S. D. Ntzouni M. Margaritis L. H.. (2010). Cell-produced alpha-synuclein is secreted in a calcium-dependent manner by exosomes and impacts neuronal survival. J. Neurosci. 30, 6838–6851. 10.1523/JNEUROSCI.5699-09.2010 20484626 PMC3842464

[B30] Emmanouilidou E. Vekrellis K. (2016). Exocytosis and spreading of normal and aberrant alpha-synuclein. Brain Pathol. 26, 398–403. 10.1111/bpa.12373 26940375 PMC8029167

[B32] Erb M. L. Moore D. J. (2020). LRRK2 and the endolysosomal system in Parkinson’s disease. J. Parkinsons Dis. 10, 1271–1291. 10.3233/JPD-202138 33044192 PMC7677880

[B33] Feng W. Zhang Y. Wang Z. Xu H. Wu T. Marshall C.. (2020). Microglia prevent beta-amyloid plaque formation in the early stage of an Alzheimer’s disease mouse model with suppression of glymphatic clearance. Alzheimers Res. Ther. 12:125. 10.1186/s13195-020-00688-1 33008458 PMC7532614

[B34] Ferraz M. J. Marques A. R. Appelman M. D. Verhoek M. Strijland A. Mirzaian M.. (2016). Lysosomal glycosphingolipid catabolism by acid ceramidase: formation of glycosphingoid bases during deficiency of glycosidases. FEBS Lett. 590, 716–725. 10.1002/1873-3468.12104 26898341

[B35] Gagliardi D. Bresolin N. Comi G. P. Corti S. (2021). Extracellular vesicles and amyotrophic lateral sclerosis: from misfolded protein vehicles to promising clinical biomarkers. Cell. Mol. Life Sci. 78, 561–572. 10.1007/s00018-020-03619-3 32803397 PMC7872995

[B36] Galvagnion C. Brown J. W. Ouberai M. M. Flagmeier P. Vendruscolo M. Buell A. K.. (2016). Chemical properties of lipids strongly affect the kinetics of the membrane-induced aggregation of alpha-synuclein. Proc. Natl. Acad. Sci. U S A 113, 7065–7070. 10.1073/pnas.1601899113 27298346 PMC4932957

[B37] Galvagnion C. Buell A. K. Meisl G. Michaels T. C. Vendruscolo M. Knowles T. P.. (2015). Lipid vesicles trigger alpha-synuclein aggregation by stimulating primary nucleation. Nat. Chem. Biol. 11, 229–234. 10.1038/nchembio.1750 25643172 PMC5019199

[B38] Ginsberg L. Rafique S. Xuereb J. H. Rapoport S. I. Gershfeld N. L. (1995). Disease and anatomic specificity of ethanolamine plasmalogen deficiency in Alzheimer’s disease brain. Brain Res. 698, 223–226. 10.1016/0006-8993(95)00931-f 8581486

[B39] Giussani P. Prinetti A. Tringali C. (2021). The role of Sphingolipids in myelination and myelin stability and their involvement in childhood and adult demyelinating disorders. J. Neurochem. 156, 403–414. 10.1111/jnc.15133 33448358

[B40] Grey M. Dunning C. J. Gaspar R. Grey C. Brundin P. Sparr E.. (2015). Acceleration of alpha-synuclein aggregation by exosomes. J. Biol. Chem. 290, 2969–2982. 10.1074/jbc.M114.585703 25425650 PMC4317028

[B41] Grossi I. Radeghieri A. Paolini L. Porrini V. Pilotto A. Padovani A.. (2021). MicroRNA34a5p expression in the plasma and in its extracellular vesicle fractions in subjects with Parkinson’s disease: an exploratory study. Int. J. Mol. Med. 47, 533–546. 10.3892/ijmm.2020.4806 33416118 PMC7797475

[B42] Grundke-Iqbal I. Iqbal K. Tung Y. C. Quinlan M. Wisniewski H. M. Binder L. I. (1986). Abnormal phosphorylation of the microtubule-associated protein tau (tau) in Alzheimer cytoskeletal pathology. Proc. Natl. Acad. Sci. U S A 83, 4913–4917. 10.1073/pnas.83.13.4913 3088567 PMC323854

[B43] Gu X. L. Long C. X. Sun L. Xie C. Lin X. Cai H. (2010). Astrocytic expression of Parkinson’s disease-related A53T alpha-synuclein causes neurodegeneration in mice. Mol. Brain 3:12. 10.1186/1756-6606-3-12 20409326 PMC2873589

[B44] Guedes L. C. Chan R. B. Gomes M. A. Conceicao V. A. Machado R. B. Soares T.. (2017). Serum lipid alterations in GBA-associated Parkinson’s disease. Parkinsonism Relat. Disord. 44, 58–65. 10.1016/j.parkreldis.2017.08.026 28890071

[B45] Guo M. Wang J. Zhao Y. Feng Y. Han S. Dong Q.. (2020). Microglial exosomes facilitate alpha-synuclein transmission in Parkinson’s disease. Brain 143, 1476–1497. 10.1093/brain/awaa090 32355963 PMC7241957

[B46] Gustafsson G. Lindstrom V. Rostami J. Nordstrom E. Lannfelt L. Bergstrom J.. (2017). Alpha-synuclein oligomer-selective antibodies reduce intracellular accumulation and mitochondrial impairment in alpha-synuclein exposed astrocytes. J. Neuroinflammation 14:241. 10.1186/s12974-017-1018-z 29228971 PMC5725978

[B47] Hait N. C. Oskeritzian C. A. Paugh S. W. Milstien S. Spiegel S. (2006). Sphingosine kinases, sphingosine 1-phosphate, apoptosis and diseases. Biochim. Biophys. Acta 1758, 2016–2026. 10.1016/j.bbamem.2006.08.007 16996023

[B48] Han X. Holtzman D. M. Mckeel D. W. Jr. Kelley J. Morris J. C. (2002). Substantial sulfatide deficiency and ceramide elevation in very early Alzheimer’s disease: potential role in disease pathogenesis. J. Neurochem. 82, 809–818. 10.1046/j.1471-4159.2002.00997.x 12358786

[B49] Hannestad J. K. Rocha S. Agnarsson B. Zhdanov V. P. Wittung-Stafshede P. Hook F. (2020). Single-vesicle imaging reveals lipid-selective and stepwise membrane disruption by monomeric alpha-synuclein. Proc. Natl. Acad. Sci. U S A 117, 14178–14186. 10.1073/pnas.1914670117 32513706 PMC7322013

[B50] Huang Y. Mahley R. W. (2014). Apolipoprotein E: structure and function in lipid metabolism, neurobiology and Alzheimer’s diseases. Neurobiol. Dis. 72, 3–12. 10.1016/j.nbd.2014.08.025 25173806 PMC4253862

[B51] Ikuno M. Yamakado H. Akiyama H. Parajuli L. K. Taguchi K. Hara J.. (2019). GBA haploinsufficiency accelerates alpha-synuclein pathology with altered lipid metabolism in a prodromal model of Parkinson’s disease. Hum. Mol. Genet. 28, 1894–1904. 10.1093/hmg/ddz030 30689867

[B52] Janas A. M. Sapon K. Janas T. Stowell M. H. Janas T. (2016). Exosomes and other extracellular vesicles in neural cells and neurodegenerative diseases. Biochim. Biophys. Acta 1858, 1139–1151. 10.1016/j.bbamem.2016.02.011 26874206

[B53] Jewett K. A. Thomas R. E. Phan C. Q. Lin B. Milstein G. Yu S.. (2021). Glucocerebrosidase reduces the spread of protein aggregation in a Drosophila melanogaster model of neurodegeneration by regulating proteins trafficked by extracellular vesicles. PLoS Genet. 17:e1008859. 10.1371/journal.pgen.1008859 33539341 PMC7888665

[B54] Kim M. J. Jeon S. Burbulla L. F. Krainc D. (2018). Acid ceramidase inhibition ameliorates alpha-synuclein accumulation upon loss of GBA1 function. Hum. Mol. Genet. 27, 1972–1988. 10.1093/hmg/ddy105 29579237 PMC6251682

[B55] Kishimoto Y. Agranoff B. W. Radin N. S. Burton R. M. (1969). Comparison of the fatty acids of lipids of subcellular brain fractions. J. Neurochem. 16, 397–404. 10.1111/j.1471-4159.1969.tb10380.x 5802640

[B56] Kjaer L. Giehm L. Heimburg T. Otzen D. (2009). The influence of vesicle size and composition on alpha-synuclein structure and stability. Biophys. J. 96, 2857–2870. 10.1016/j.bpj.2008.12.3940 19348768 PMC2711279

[B57] Kurzawa-Akanbi M. Tammireddy S. Fabrik I. Gliaudelyte L. Doherty M. K. Heap R.. (2021). Altered ceramide metabolism is a feature in the extracellular vesicle-mediated spread of alpha-synuclein in Lewy body disorders. Acta Neuropathol. 142, 961–984. 10.1007/s00401-021-02367-3 34514546 PMC8568874

[B58] Laulagnier K. Javalet C. Hemming F. J. Chivet M. Lachenal G. Blot B.. (2018). Amyloid precursor protein products concentrate in a subset of exosomes specifically endocytosed by neurons. Cell. Mol. Life Sci. 75, 757–773. 10.1007/s00018-017-2664-0 28956068 PMC11105273

[B59] Lawson C. Kovacs D. Finding E. Ulfelder E. Luis-Fuentes V. (2017). Extracellular vesicles: evolutionarily conserved mediators of intercellular communication. Yale J. Biol. Med. 90, 481–491. 28955186 PMC5612190

[B60] Lee H. J. Suk J. E. Patrick C. Bae E. J. Cho J. H. Rho S.. (2010). Direct transfer of alpha-synuclein from neuron to astroglia causes inflammatory responses in synucleinopathies. J. Biol. Chem. 285, 9262–9272. 10.1074/jbc.M109.081125 20071342 PMC2838344

[B61] Lindstrom V. Gustafsson G. Sanders L. H. Howlett E. H. Sigvardson J. Kasrayan A.. (2017). Extensive uptake of alpha-synuclein oligomers in astrocytes results in sustained intracellular deposits and mitochondrial damage. Mol. Cell. Neurosci. 82, 143–156. 10.1016/j.mcn.2017.04.009 28450268

[B62] Liu L. Drouet V. Wu J. W. Witter M. P. Small S. A. Clelland C.. (2012). Trans-synaptic spread of tau pathology *in vivo*. PLoS One 7:e31302. 10.1371/journal.pone.0031302 22312444 PMC3270029

[B63] Loria F. Vargas J. Y. Bousset L. Syan S. Salles A. Melki R.. (2017). alpha-Synuclein transfer between neurons and astrocytes indicates that astrocytes play a role in degradation rather than in spreading. Acta Neuropathol. 134, 789–808. 10.1007/s00401-017-1746-2 28725967

[B64] Lusardi T. A. Phillips J. I. Wiedrick J. T. Harrington C. A. Lind B. Lapidus J. A.. (2017). MicroRNAs in human cerebrospinal fluid as biomarkers for Alzheimer’s disease. J. Alzheimers Dis. 55, 1223–1233. 10.3233/JAD-160835 27814298 PMC5587208

[B65] Mahley R. W. (1988). Apolipoprotein E: cholesterol transport protein with expanding role in cell biology. Science 240, 622–630. 10.1126/science.3283935 3283935

[B66] Marsh J. Alifragis P. (2018). Synaptic dysfunction in Alzheimer’s disease: the effects of amyloid beta on synaptic vesicle dynamics as a novel target for therapeutic intervention. Neural Regen. Res. 13, 616–623. 10.4103/1673-5374.230276 29722304 PMC5950662

[B67] Martini-Stoica H. Cole A. L. Swartzlander D. B. Chen F. Wan Y. W. Bajaj L.. (2018). TFEB enhances astroglial uptake of extracellular tau species and reduces tau spreading. J. Exp. Med. 215, 2355–2377. 10.1084/jem.20172158 30108137 PMC6122971

[B68] Masters C. L. Simms G. Weinman N. A. Multhaup G. Mcdonald B. L. Beyreuther K. (1985). Amyloid plaque core protein in Alzheimer disease and Down syndrome. Proc. Natl. Acad. Sci. U S A 82, 4245–4249. 10.1073/pnas.82.12.4245 3159021 PMC397973

[B69] Mate De Gerando A. D’orange M. Augustin E. Josephine C. Auregan G. Gaudin-Guerif M.. (2021). Neuronal tau species transfer to astrocytes and induce their loss according to tau aggregation state. Brain 144, 1167–1182. 10.1093/brain/awab011 33842937

[B70] McInnes J. Wierda K. Snellinx A. Bounti L. Wang Y. C. Stancu I. C.. (2018). Synaptogyrin-3 mediates presynaptic dysfunction induced by Tau. Neuron 97, 823–835.e8. 10.1016/j.neuron.2018.01.022 29398363

[B71] Mencarelli C. Martinez-Martinez P. (2013). Ceramide function in the brain: when a slight tilt is enough. Cell. Mol. Life Sci. 70, 181–203. 10.1007/s00018-012-1038-x 22729185 PMC3535405

[B72] Menck K. Sonmezer C. Worst T. S. Schulz M. Dihazi G. H. Streit F.. (2017). Neutral sphingomyelinases control extracellular vesicles budding from the plasma membrane. J. Extracellular Vesicles 6:1378056. 10.1080/20013078.2017.1378056 29184623 PMC5699186

[B73] Mesa-Herrera F. Taoro-Gonzalez L. Valdes-Baizabal C. Diaz M. Marin R. (2019). Lipid and lipid raft alteration in aging and neurodegenerative diseases: a window for the development of new biomarkers. Int. J. Mol. Sci. 20:3810. 10.3390/ijms20153810 31382686 PMC6696273

[B74] Meyer-Luehmann M. Spires-Jones T. L. Prada C. Garcia-Alloza M. De Calignon A. Rozkalne A.. (2008). Rapid appearance and local toxicity of amyloid-beta plaques in a mouse model of Alzheimer’s disease. Nature 451, 720–724. 10.1038/nature06616 18256671 PMC3264491

[B75] Middleton E. R. Rhoades E. (2010). Effects of curvature and composition on alpha-synuclein binding to lipid vesicles. Biophys. J. 99, 2279–2288. 10.1016/j.bpj.2010.07.056 20923663 PMC3042580

[B76] Mielke M. M. Bandaru V. V. Haughey N. J. Xia J. Fried L. P. Yasar S.. (2012). Serum ceramides increase the risk of Alzheimer disease: the Women’s Health and Aging Study II. Neurology 79, 633–641. 10.1212/WNL.0b013e318264e380 22815558 PMC3414665

[B77] Mielke M. M. Haughey N. J. Bandaru V. V. Schech S. Carrick R. Carlson M. C.. (2010). Plasma ceramides are altered in mild cognitive impairment and predict cognitive decline and hippocampal volume loss. Alzheimers Dement. 6, 378–385. 10.1016/j.jalz.2010.03.014 20813340 PMC2933928

[B78] Mielke M. M. Maetzler W. Haughey N. J. Bandaru V. V. Savica R. Deuschle C.. (2013). Plasma ceramide and glucosylceramide metabolism is altered in sporadic Parkinson’s disease and associated with cognitive impairment: a pilot study. PLoS One 8:e73094. 10.1371/journal.pone.0073094 24058461 PMC3776817

[B79] Minakaki G. Menges S. Kittel A. Emmanouilidou E. Schaeffner I. Barkovits K.. (2018). Autophagy inhibition promotes SNCA/alpha-synuclein release and transfer *via* extracellular vesicles with a hybrid autophagosome-exosome-like phenotype. Autophagy 14, 98–119. 10.1080/15548627.2017.1395992 29198173 PMC5846507

[B80] Miranda A. M. Lasiecka Z. M. Xu Y. Neufeld J. Shahriar S. Simoes S.. (2018). Neuronal lysosomal dysfunction releases exosomes harboring APP C-terminal fragments and unique lipid signatures. Nat. Commun. 9:291. 10.1038/s41467-017-02533-w 29348617 PMC5773483

[B81] Miyoshi E. Bilousova T. Melnik M. Fakhrutdinov D. Poon W. W. Vinters H. V.. (2021). Exosomal tau with seeding activity is released from Alzheimer’s disease synapses and seeding potential is associated with amyloid beta. Lab. Invest. 101, 1605–1617. 10.1038/s41374-021-00644-z 34462532 PMC8590975

[B82] Morel E. Chamoun Z. Lasiecka Z. M. Chan R. B. Williamson R. L. Vetanovetz C.. (2013). Phosphatidylinositol-3-phosphate regulates sorting and processing of amyloid precursor protein through the endosomal system. Nat. Commun. 4:2250. 10.1038/ncomms3250 23907271 PMC3905799

[B83] Mulder S. D. Nielsen H. M. Blankenstein M. A. Eikelenboom P. Veerhuis R. (2014). Apolipoproteins E and J interfere with amyloid-beta uptake by primary human astrocytes and microglia *in vitro*. Glia 62, 493–503. 10.1002/glia.22619 24446231

[B84] Murphy K. E. Gysbers A. M. Abbott S. K. Tayebi N. Kim W. S. Sidransky E.. (2014). Reduced glucocerebrosidase is associated with increased alpha-synuclein in sporadic Parkinson’s disease. Brain 137, 834–848. 10.1093/brain/awt367 24477431 PMC3927701

[B85] Nalls M. A. Duran R. Lopez G. Kurzawa-Akanbi M. Mckeith I. G. Chinnery P. F.. (2013). A multicenter study of glucocerebrosidase mutations in dementia with Lewy bodies. JAMA Neurol. 70, 727–735. 10.1001/jamaneurol.2013.1925 23588557 PMC3841974

[B86] Narasimhan S. Changolkar L. Riddle D. M. Kats A. Stieber A. Weitzman S. A.. (2020). Human tau pathology transmits glial tau aggregates in the absence of neuronal tau. J. Exp. Med. 217:e20190783. 10.1084/jem.20190783 31826239 PMC7041709

[B87] Nath S. Agholme L. Kurudenkandy F. R. Granseth B. Marcusson J. Hallbeck M. (2012). Spreading of neurodegenerative pathology *via* neuron-to-neuron transmission of beta-amyloid. J. Neurosci. 32, 8767–8777. 10.1523/JNEUROSCI.0615-12.2012 22745479 PMC6622335

[B88] Neudorfer O. Giladi N. Elstein D. Abrahamov A. Turezkite T. Aghai E.. (1996). Occurrence of Parkinson’s syndrome in type I Gaucher disease. QJM 89, 691–694. 10.1093/qjmed/89.9.691 8917744

[B89] Neumann H. Kotter M. R. Franklin R. J. (2009). Debris clearance by microglia: an essential link between degeneration and regeneration. Brain 132, 288–295. 10.1093/brain/awn109 18567623 PMC2640215

[B90] Ngolab J. Trinh I. Rockenstein E. Mante M. Florio J. Trejo M.. (2017). Brain-derived exosomes from dementia with Lewy bodies propagate alpha-synuclein pathology. Acta Neuropathol. Commun. 5:46. 10.1186/s40478-017-0445-5 28599681 PMC5466770

[B91] Nilson A. N. English K. C. Gerson J. E. Barton Whittle T. Nicolas Crain C. Xue J.. (2017). Tau oligomers associate with inflammation in the brain and retina of tauopathy mice and in neurodegenerative diseases. J. Alzheimers Dis. 55, 1083–1099. 10.3233/JAD-160912 27716675 PMC5147514

[B92] Nortley R. Attwell D. (2017). Control of brain energy supply by astrocytes. Curr. Opin. Neurobiol. 47, 80–85. 10.1016/j.conb.2017.09.012 29054039

[B93] O’brien J. S. Sampson E. L. (1965). Lipid composition of the normal human brain: gray matter, white matter and myelin. J. Lipid Res. 6, 537–544. 5865382

[B94] Papadopoulos V. E. Nikolopoulou G. Antoniadou I. Karachaliou A. Arianoglou G. Emmanouilidou E.. (2018). Modulation of beta-glucocerebrosidase increases alpha-synuclein secretion and exosome release in mouse models of Parkinson’s disease. Hum. Mol. Genet. 27, 1696–1710. 10.1093/hmg/ddy075 29547959

[B95] Peng K. Y. Perez-Gonzalez R. Alldred M. J. Goulbourne C. N. Morales-Corraliza J. Saito M.. (2019). Apolipoprotein E4 genotype compromises brain exosome production. Brain 142, 163–175. 10.1093/brain/awy289 30496349 PMC6308312

[B96] Perea J. R. Lopez E. Diez-Ballesteros J. C. Avila J. Hernandez F. Bolos M. (2019). Extracellular monomeric tau is internalized by astrocytes. Front. Neurosci. 13:442. 10.3389/fnins.2019.00442 31118883 PMC6504834

[B97] Phelps C. H. (1972). The development of glio-vascular relationships in the rat spinal cord. An electron microscopic study. Z. Zellforsch. Mikrosk. Anat. 128, 555–563. 10.1007/BF00306988 4553990

[B98] Prada I. Gabrielli M. Turola E. Iorio A. D’arrigo G. Parolisi R.. (2018). Glia-to-neuron transfer of miRNAs *via* extracellular vesicles: a new mechanism underlying inflammation-induced synaptic alterations. Acta Neuropathol. 135, 529–550. 10.1007/s00401-017-1803-x 29302779 PMC5978931

[B99] Pranke I. M. Morello V. Bigay J. Gibson K. Verbavatz J. M. Antonny B.. (2011). alpha-Synuclein and ALPS motifs are membrane curvature sensors whose contrasting chemistry mediates selective vesicle binding. J. Cell Biol. 194, 89–103. 10.1083/jcb.201011118 21746853 PMC3135411

[B100] Qi G. Mi Y. Shi X. Gu H. Brinton R. D. Yin F. (2021). ApoE4 impairs neuron-astrocyte coupling of fatty acid metabolism. Cell Rep. 34:108572. 10.1016/j.celrep.2020.108572 33406436 PMC7837265

[B101] Rajendran L. Honsho M. Zahn T. R. Keller P. Geiger K. D. Verkade P.. (2006). Alzheimer’s disease beta-amyloid peptides are released in association with exosomes. Proc. Natl. Acad. Sci. U S A 103, 11172–11177. 10.1073/pnas.0603838103 16837572 PMC1544060

[B102] Richetin K. Steullet P. Pachoud M. Perbet R. Parietti E. Maheswaran M.. (2020). Tau accumulation in astrocytes of the dentate gyrus induces neuronal dysfunction and memory deficits in Alzheimer’s disease. Nat. Neurosci. 23, 1567–1579. 10.1038/s41593-020-00728-x 33169029

[B103] Sackmann V. Sinha M. S. Sackmann C. Civitelli L. Bergstrom J. Ansell-Schultz A.. (2019). Inhibition of nSMase2 reduces the transfer of oligomeric alpha-synuclein irrespective of hypoxia. Front. Mol. Neurosci. 12:200. 10.3389/fnmol.2019.00200 31555088 PMC6724746

[B104] Saman S. Kim W. Raya M. Visnick Y. Miro S. Saman S.. (2012). Exosome-associated tau is secreted in tauopathy models and is selectively phosphorylated in cerebrospinal fluid in early Alzheimer disease. J. Biol. Chem. 287, 3842–3849. 10.1074/jbc.M111.277061 22057275 PMC3281682

[B105] Sardar Sinha M. Ansell-Schultz A. Civitelli L. Hildesjo C. Larsson M. Lannfelt L.. (2018). Alzheimer’s disease pathology propagation by exosomes containing toxic amyloid-beta oligomers. Acta Neuropathol. 136, 41–56. 10.1007/s00401-018-1868-1 29934873 PMC6015111

[B106] Sardi S. P. Viel C. Clarke J. Treleaven C. M. Richards A. M. Park H.. (2017). Glucosylceramide synthase inhibition alleviates aberrations in synucleinopathy models. Proc. Natl. Acad. Sci. U S A 114, 2699–2704. 10.1073/pnas.1616152114 28223512 PMC5347608

[B107] Satoi H. Tomimoto H. Ohtani R. Kitano T. Kondo T. Watanabe M.. (2005). Astroglial expression of ceramide in Alzheimer’s disease brains: a role during neuronal apoptosis. Neuroscience 130, 657–666. 10.1016/j.neuroscience.2004.08.056 15590150

[B108] Savica R. Murray M. E. Persson X. M. Kantarci K. Parisi J. E. Dickson D. W.. (2016). Plasma sphingolipid changes with autopsy-confirmed Lewy Body or Alzheimer’s pathology. Alzheimers Dement. (Amst) 3, 43–50. 10.1016/j.expneurol.2021.113929 27152320 PMC4852484

[B109] Shahmoradian S. H. Lewis A. J. Genoud C. Hench J. Moors T. E. Navarro P. P.. (2019). Lewy pathology in Parkinson’s disease consists of crowded organelles and lipid membranes. Nat. Neurosci. 22, 1099–1109. 10.1038/s41593-019-0423-2 31235907

[B110] Sharon R. Bar-Joseph I. Frosch M. P. Walsh D. M. Hamilton J. A. Selkoe D. J. (2003). The formation of highly soluble oligomers of alpha-synuclein is regulated by fatty acids and enhanced in Parkinson’s disease. Neuron 37, 583–595. 10.1016/s0896-6273(03)00024-2 12597857

[B111] Shen D. Wang X. Xu H. (2011). Pairing phosphoinositides with calcium ions in endolysosomal dynamics: phosphoinositides control the direction and specificity of membrane trafficking by regulating the activity of calcium channels in the endolysosomes. Bioessays 33, 448–457. 10.1002/bies.201000152 21538413 PMC3107950

[B112] Sidransky E. Nalls M. A. Aasly J. O. Aharon-Peretz J. Annesi G. Barbosa E. R.. (2009). Multicenter analysis of glucocerebrosidase mutations in Parkinson’s disease. N. Engl. J. Med. 361, 1651–1661. 10.1056/NEJMoa0901281 19846850 PMC2856322

[B113] Skotland T. Sandvig K. Llorente A. (2017). Lipids in exosomes: current knowledge and the way forward. Prog. Lipid Res. 66, 30–41. 10.1016/j.plipres.2017.03.001 28342835

[B114] Sollvander S. Nikitidou E. Brolin R. Soderberg L. Sehlin D. Lannfelt L.. (2016). Accumulation of amyloid-beta by astrocytes result in enlarged endosomes and microvesicle-induced apoptosis of neurons. Mol. Neurodegener. 11:38. 10.1186/s13024-016-0098-z 27176225 PMC4865996

[B115] Spillantini M. G. Schmidt M. L. Lee V. M. Trojanowski J. Q. Jakes R. Goedert M. (1997). Alpha-synuclein in Lewy bodies. Nature 388, 839–840. 10.1038/42166 9278044

[B116] Stahl A. L. Johansson K. Mossberg M. Kahn R. Karpman D. (2019). Exosomes and microvesicles in normal physiology, pathophysiology and renal diseases. Pediatr. Nephrol. 34, 11–30. 10.1007/s00467-017-3816-z 29181712 PMC6244861

[B117] Stalder D. Gershlick D. C. (2020). Direct trafficking pathways from the Golgi apparatus to the plasma membrane. Semin. Cell Dev. Biol. 107, 112–125. 10.1016/j.semcdb.2020.04.001 32317144 PMC7152905

[B118] Stobart J. L. Anderson C. M. (2013). Multifunctional role of astrocytes as gatekeepers of neuronal energy supply. Front. Cell. Neurosci. 7:38. 10.3389/fncel.2013.00038 23596393 PMC3622037

[B119] Stuendl A. Kunadt M. Kruse N. Bartels C. Moebius W. Danzer K. M.. (2016). Induction of alpha-synuclein aggregate formation by CSF exosomes from patients with Parkinson’s disease and dementia with Lewy bodies. Brain 139, 481–494. 10.1093/brain/awv346 26647156 PMC4805087

[B120] Sudhof T. C. Rizo J. (2011). Synaptic vesicle exocytosis. Cold Spring Harb. Perspect. Biol. 3:a005637. 10.1101/cshperspect.a005637 22026965 PMC3225952

[B121] Sugiura Y. Ikeda K. Nakano M. (2015). High membrane curvature enhances binding, conformational changes and fibrillation of amyloid-beta on lipid bilayer surfaces. Langmuir 31, 11549–11557. 10.1021/acs.langmuir.5b03332 26474149

[B122] Taniguchi M. Okazaki T. (2020). Ceramide/sphingomyelin rheostat regulated by sphingomyelin synthases and chronic diseases in murine models. J. Lipid Atheroscler. 9, 380–405. 10.12997/jla.2020.9.3.380 33024732 PMC7521967

[B123] Tayebi N. Parisiadou L. Berhe B. Gonzalez A. N. Serra-Vinardell J. Tamargo R. J.. (2017). Glucocerebrosidase haploinsufficiency in A53T alpha-synuclein mice impacts disease onset and course. Mol. Genet. Metab. 122, 198–208. 10.1016/j.ymgme.2017.11.001 29173981 PMC6007972

[B124] Thery C. Zitvogel L. Amigorena S. (2002). Exosomes: composition, biogenesis and function. Nat. Rev. Immunol. 2, 569–579. 10.1038/nri855 12154376

[B125] Thomas R. E. Vincow E. S. Merrihew G. E. Maccoss M. J. Davis M. Y. Pallanck L. J. (2018). Glucocerebrosidase deficiency promotes protein aggregation through dysregulation of extracellular vesicles. PLoS Genet. 14:e1007694. 10.1371/journal.pgen.1007694 30256786 PMC6175534

[B126] Trajkovic K. Hsu C. Chiantia S. Rajendran L. Wenzel D. Wieland F.. (2008). Ceramide triggers budding of exosome vesicles into multivesicular endosomes. Science 319, 1244–1247. 10.1126/science.1153124 18309083

[B127] Tsunemi T. Ishiguro Y. Yoroisaka A. Valdez C. Miyamoto K. Ishikawa K.. (2020). Astrocytes protect human dopaminergic neurons from alpha-synuclein accumulation and propagation. J. Neurosci. 40, 8618–8628. 10.1523/JNEUROSCI.0954-20.2020 33046546 PMC7643299

[B128] Tu P. H. Galvin J. E. Baba M. Giasson B. Tomita T. Leight S.. (1998). Glial cytoplasmic inclusions in white matter oligodendrocytes of multiple system atrophy brains contain insoluble alpha-synuclein. Ann. Neurol. 44, 415–422. 10.1002/ana.410440324 9749615

[B129] Vogel J. W. Iturria-Medina Y. Strandberg O. T. Smith R. Levitis E. Evans A. C.. (2020). Spread of pathological tau proteins through communicating neurons in human Alzheimer’s disease. Nat. Commun. 11:2612. 10.1038/s41467-020-15701-2 32457389 PMC7251068

[B134] Wang Y. Balaji V. Kaniyappan S. Kruger L. Irsen S. Tepper K.. (2017). The release and trans-synaptic transmission of Tau *via* exosomes. Mol. Neurodegener. 12:5. 10.1186/s13024-016-0143-y 28086931 PMC5237256

[B130] Wang G. Bieberich E. (2018). Sphingolipids in neurodegeneration (with focus on ceramide and S1P). Adv. Biol. Regul. 70, 51–64. 10.1016/j.jbior.2018.09.013 30287225 PMC6251739

[B131] Wang G. Dinkins M. He Q. Zhu G. Poirier C. Campbell A.. (2012). Astrocytes secrete exosomes enriched with proapoptotic ceramide and prostate apoptosis response 4 (PAR-4): potential mechanism of apoptosis induction in Alzheimer disease (AD). J. Biol. Chem. 287, 21384–21395. 10.1038/s41467-020-15701-2 22532571 PMC3375560

[B132] Wang G. Silva J. Dasgupta S. Bieberich E. (2008). Long-chain ceramide is elevated in presenilin 1 (PS1M146V) mouse brain and induces apoptosis in PS1 astrocytes. Glia 56, 449–456. 10.1002/glia.20626 18205190

[B133] Wang W. Zhu N. Yan T. Shi Y. N. Chen J. Zhang C. J.. (2020). The crosstalk: exosomes and lipid metabolism. Cell Commun. Signal. 18:119. 10.1186/s12964-020-00581-2 32746850 PMC7398059

[B135] Weeratunga S. Paul B. Collins B. M. (2020). Recognising the signals for endosomal trafficking. Curr. Opin. Cell Biol. 65, 17–27. 10.1016/j.ceb.2020.02.005 32155566

[B136] Winder-Rhodes S. E. Evans J. R. Ban M. Mason S. L. Williams-Gray C. H. Foltynie T.. (2013). Glucocerebrosidase mutations influence the natural history of Parkinson’s disease in a community-based incident cohort. Brain 136, 392–399. 10.1093/brain/aws318 23413260

[B137] Wu L. G. Hamid E. Shin W. Chiang H. C. (2014). Exocytosis and endocytosis: modes, functions and coupling mechanisms. Annu. Rev. Physiol. 76, 301–331. 10.1146/annurev-physiol-021113-170305 24274740 PMC4880020

[B138] Xia Y. Zhang G. Han C. Ma K. Guo X. Wan F.. (2019). Microglia as modulators of exosomal alpha-synuclein transmission. Cell Death Dis. 10:174. 10.1038/s41419-019-1404-9 30787269 PMC6382842

[B139] Yuyama K. Sun H. Mitsutake S. Igarashi Y. (2012). Sphingolipid-modulated exosome secretion promotes clearance of amyloid-beta by microglia. J. Biol. Chem. 287, 10977–10989. 10.1074/jbc.M111.324616 22303002 PMC3322859

[B140] Yuyama K. Sun H. Sakai S. Mitsutake S. Okada M. Tahara H.. (2014). Decreased amyloid-beta pathologies by intracerebral loading of glycosphingolipid-enriched exosomes in Alzheimer model mice. J. Biol. Chem. 289, 24488–24498. 10.1074/jbc.M114.577213 25037226 PMC4148874

[B141] Zhang J. Li S. Li L. Li M. Guo C. Yao J.. (2015). Exosome and exosomal microRNA: trafficking, sorting and function. Genomics Proteomics Bioinformatics 13, 17–24. 10.1016/j.gpb.2015.02.001 25724326 PMC4411500

[B142] Zhou L. Mcinnes J. Wierda K. Holt M. Herrmann A. G. Jackson R. J.. (2017). Tau association with synaptic vesicles causes presynaptic dysfunction. Nat. Commun. 8:15295. 10.1038/ncomms15295 28492240 PMC5437271

